# Supplementary material for: The impact of brominated flame retardants exposure on serum total bilirubin: A cross-sectional analysis
Source: PLoS One. 2025 Mar 28;20(3):e0320523. doi: 10.1371/journal.pone.0320523 (PMC11952259; doi:10.1371/journal.pone.0320523)
Supplement: Attachment — Submitted filename: Reviewer Comments.docx [file pone.0320523.s001.docx]

**Comments for the Research paper entitled ‘The Impact of Brominated Flame Retardants Exposure on Serum Total Bilirubin: A Cross-Sectional Analysis’**

1. What is the significance of this study in the context of comprehending the relationship between BFRs and total bilirubin (TB) levels?
2. Provide additional information regarding the brominated flame retardants (BFRs) and the reasons for their classification as hazardous environmental pollutants in

the introduction section.

3. In this investigation, how were total bilirubin (TB) levels classified?
4. Check the discrepancy between the years of NHANES data mentioned in the

abstract (2007–2016) and the methodology (2005–2016).

5. The methodology for measuring BFRs and TB levels is unclear. Provide/cite the relevant references or links to NHANES protocols to enhance clarity.
6. How did adjusting for confounding factors affect the results?
7. What types of correlations (e.g., U-shaped, inverted J shaped) were observed between specific BFRs and TB levels and what is the significance of these findings?
8. What was the impact of the stratified analysis by gender and age on the results?
9. In what manner were combined exposures to BFRs assessed in this study?
10. What is the significance of stratified analysis by age and gender in understanding the correlation between BFRs and TB levels?
11. In the discussion section, provide additional information regarding the significant PBDEs observed in conditions such as metabolic syndrome, COPD and neurological disorders.
